# Altered Baseline Brain Activity with 72 h of Simulated Microgravity – Initial Evidence from Resting-State fMRI

**DOI:** 10.1371/journal.pone.0052558

**Published:** 2012-12-21

**Authors:** Yang Liao, Jinsong Zhang, Zhiping Huang, Yibin Xi, Qianru Zhang, Tianli Zhu, Xufeng Liu

**Affiliations:** 1 Department of Psychology, School of Aerospace Medicine, Fourth Military Medical University, Xi’an, Shaanxi, China; 2 Department of Radiology, Xijing Hospital, Fourth Military Medical University, Xi’an, Shaanxi, China; Hangzhou Normal University, China

## Abstract

To provide the basis and reference to further insights into the neural activity of the human brain in a microgravity environment, we discuss the amplitude changes of low-frequency brain activity fluctuations using a simulated microgravity model. Twelve male participants between 24 and 31 years old received resting-state fMRI scans in both a normal condition and after 72 hours in a −6° head down tilt (HDT). A paired sample t-test was used to test the amplitude differences of low-frequency brain activity fluctuations between these two conditions. With 72 hours in a −6° HDT, the participants showed a decreased amplitude of low-frequency fluctuations in the left thalamus compared with the normal condition (a combined threshold of *P*<0.005 and a minimum cluster size of 351 mm^3^ (13 voxels), which corresponded with the corrected threshold of *P*<0.05 determined by AlphaSim). Our findings indicate that a gravity change-induced redistribution of body fluid may disrupt the function of the left thalamus in the resting state, which may contribute to reduced motor control abilities and multiple executive functions in astronauts in a microgravity environment.

## Introduction

Maintaining an astronaut’s performance at an optimal level is of importance to space medical and psychological researchers. It is commonly known that an astronaut’s performance might be impaired because of harmful factors, such as radiation, noise, a changed circadian rhythm, and microgravity [1]. Among these factors, microgravity is the most significant difference between space and earth environments; therefore, astronauts’ performance degradation caused by microgravity has been a common focus among researchers in different fields. Previous studies have reported that astronauts experience various performance degradations in microgravity. Newman et al. reported microgravity effects on manual control movements, which indicated a disruption in fine motor control [2]. In two studies of microgravity performed by Manzey et al., obvious decrements in visual motor function and tracking performance were observed [3], [4]. Additionally, several studies indicated that planning movements and goal-directed actions, which require fine motor control, are also impaired in microgravity [2], [5], [6], [7], [8], [9]. These degradations in behavioral and cognitive functions may cause potential risks in space exploration. To reduce the possibility of an accident, determining the mechanisms of behavioral and cognitive performance degradations in microgravity and the corresponding countermeasures are of great significance and must be challengeable tasks for aerospace medicine researchers.

Previous studies have demonstrated that effective behavior depends on the dynamic integration of sensory, motor, and cognitive functions on multiple spatial and temporal scales [10]; therefore, the brain naturally plays an important role as an optimal function integration system for performance maintenance. To clarify the mechanisms of performance degradations in microgravity, it is necessary to determine the conclusive evidence of altered brain activities in microgravity. Some physiology researchers have attempted to solve this problem using physiological methods [11], [12], [13]. The authors of these physiology studies successfully observed changed volumes of cerebrospinal fluid, cerebral blood flow, and altered intracranial pressure in astronauts in a weightless environment. The authors determined that these variations were caused by the redistribution of an individual’s body fluid toward the head, which they called the microgravity effect. Unfortunately, because of the limitations of their research, the authors could not clarify how the physiological changes in the brain contributed to the observed performance degradations.

Some researchers have attempted to determine how brain physiological changes contribute to a decrease in performance using new methods [14], [15], [16], [17]. The authors of these studies performed pioneering work using an electroencephalograph (EEG) to directly record the altered brain electrical activity to provide more reasonable explanations for performance degradations in microgravity. Evidence from Cheron and colleagues suggests that altered alpha and mu rhythms, as measured by an EEG, were observed when the participants were exposed to a microgravity environment, which indicated a change in the arousal level of the brain that could affect cognitive function and behavior performance [14]. Schneider et al. observed an inhibited beta-2 EEG in the inferior temporal gyrus in microgravity, which may reflect changed emotion processes [17]. These findings were encouraging; however, the authors could not locate the precise brain area that contributes to definite performance degradation because of the poor resolution of the EEG in space.

Fortunately, with the development of new experimental techniques, the application of high spatial resolution functional magnetic resonance imaging (fMRI) could complement the limitations of EEG recordings [18], [19]. Particularly, resting-state fMRI technology could provide more stable and effective evidence in the exploration of individual brain activities; thus, resting-state fMRI is a suitable technique to complete the work left by EEG. Of the techniques used with resting-state fMRI, the amplitude of low-frequency (0.01–0.08 Hz) fluctuation (ALFF) is a useful method to observe altered brain activities [20]. Amount of previous studies had made it clear that ALFF could reflect the extent of spontaneous neuronal activity (SNA). Initial study by Biswal et al. confirmed that the ALFF is higher in gray matter than in white matter [21]. Later, by using the power spectrum method, Kiviniemi et al. reported that amplitude of low frequency fluctuation in the visual cortex at approximately 0.034 Hz may be related to regional spontaneous neuronal activity [22]. In recent years, more and more ALFF studies confirmed that the changed ALFF may be reflect hyperfunction or hypofunction in correlated brain areas [23], [24], [25], [26], [27]. Thus it can be seen that by using the indicator called ALFF, which reveals the spontaneous activity of each voxel of the brain, we could learn the strength of the brain activities in a quantitative manner.

Although resting-state fMRI is a useful technique in medical research, its use in a real microgravity environment is nearly impossible because the MRI scanner is large and requires a stable work environment. Fortunately, −6° head down tilt (HDT) bed rest is a generally accepted technology in studies of microgravity among space science researchers that can simulate the microgravity effect of an individual’s body fluid redistribution toward the head [28], thus making it possible to explore the effects of microgravity on individual brain activities while on Earth.

To the best of our knowledge, no previous study has examined the altered brain activities in microgravity with a resting-state fMRI. In the current study, we combined resting state fMRI technology and −6° HDT head down tilt bed rest technology to determine ALFF differences with and without 72 hours in −6° HDT bed rest. The results of this study may support the findings of previous studies and serve as a foundation for additional studies of individual altered brain activities in a microgravity environment.

## Materials and Methods

### Participants

Sixteen healthy participants were recruited for the present study, and four participants were excluded from the analyses because of excessive head motion (more than 2 mm or 2 degree on any axis). Altogether, the data of the 12 participants met the above-mentioned criteria. Because most astronauts are males, the selected participants were normal adult males. The valid participants’ mean age was 26 years, with a range from 24 to 31 years. All participants were right-handed, as measured by the Handedness Questionnaire [29]. The participants reported no history of neurological injury, genetic mental disorders or substance abuse. With a high-resolution T1- and T2-weighted MRI examination, no participant was observed to have significant pathological changes in their brain. The study was approved by the Ethical Committee of the Fourth Military Medical University and all participants provided their written informed consent before the experiment.

### Design

Pair comparisons of the participants’ resting-state brain activities between normal and simulated microgravity conditions were applied in the present study. The normal condition collected data of the participants’ resting-state brain activities under normal gravity. The simulated microgravity condition collected data of the participants’ resting-state brain activities after 72 hours in −6° HDT bed rest. During the HDT bed rest, adequate water and food were supplied, but the participants’ heads were prevented from moving from the bed to keep the redistribution of the individual’s body fluid toward the head. The order of the fMRI scans for the two conditions were balanced to avoid the fMRI scanning order becoming an interference factor. One-half of the participants’ data in the simulated microgravity condition was collected ten days after the data collection in the normal condition, whereas the remaining participants’ data collection order was reversed. Moreover, to avoid the interference of biological rhythms, data collections were all performed at identical times during the day.

### Data Acquisitions

Image data were collected by an experienced radiologist with a 3T Magnetom Trio Tim scanner in the radiology department of the Xijing Hospital of the Fourth Military Medical University. The participants were instructed to lie flat on the scanning platform and place their head in the assigned location. Sponge earplugs were used to plug the participant’s ears to minimize noise interference, and foam padding was used to restrict head motion. A standard 16-channel head coil was used to collect data. The participants were instructed to keep their head motionless, to remain calm with their eyes closed and to not think of anything in particular. The scanning sequence and parameters used the following settings: 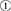 anatomical images of each location were T1-weighted images, which used the spin echo method (SE): TR = 500 ms, TE = 15 ms, flip angle (FA) = 90°, field of view (FOV) = 220 mm×220 mm, matrix = 256×144, and slice thickness = 4 mm with no gap; 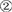 a MPRAGE sequence was used for the high-definition three-dimensional T1-weighted images of the whole brain: TR = 2530 ms, TE = 3.39 ms, inversion time = 1100 ms, FA = 7°, FOV = 256 mm×256 mm, matrix = 256×192, slice thickness = 1.33 mm with no gap, and 128 slices; 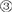 and an echo planner imaging sequence was used to acquire functional images: TR = 2000 ms, TE = 30 ms, FA = 90°, FOV = 200 mm×200 mm, matrix = 64×64, slice thickness = 3 mm, gap = 0.6 mm, and 33 slices. This section of the scan contained 240 time points and lasted for 8 minutes.

### Data Preprocessing

The Data Processing Assistant for Resting-State fMRI (DPARSF, http://www.restfmri.net/forum/DPARSF) V2.0 was used for data preprocessing. The preprocessing steps were as follows. Initially, the data format of the functional images was transformed from DICOM to NIFTI. Second, because the participants required time to adjust, the initial ten time points were discarded, and 230 time points remained. Third, the left functional images were slice-time corrected and aligned with the initial image of each session for motion correction. Four participants were excluded from the analyses because of excessive head motion (more than 2 mm or 2 degree on any axis). Fourth, the data were then spatially normalized with the Montreal Neurological Institute (MNI) template (resampling voxel size = 3×3×3 mm^3^) in the Resting-State fMRI Data Analysis Toolkit (REST, www.restfmri.net) v1.0 [30]. Fifth, the linear trend was removed, and the fMRI data were temporally band-pass filtered (0.01≤ f ≤0.08 Hz) to reduce the low-frequency drift and physiological high-frequency respiratory and cardiac noise. The resulting data were then spatially smoothed (4 mm FWHM Gaussian kernel).

### ALFF Analysis

The REST was used to perform the ALFF analysis. The procedure is similar to that used in previous studies [31]. The filtered time series was transformed to a frequency domain using a fast Fourier transform (FFT) (parameters: taper percent = 0, FFT length = shortest), and the power spectrum was obtained. Because the power of a given frequency is proportional to the square of the amplitude of the frequency component, its square root was calculated at each frequency of the power spectrum, and then the averaged square root was obtained across 0.01–0.08 Hz for each voxel. This averaged square root was considered the ALFF. For standardization purposes, the ALFF of each voxel was divided by the global mean ALFF value within a default brain mask in REST.

### Statistics

To explore the ALFF differences between the normal and simulated microgravity conditions, a paired-sample t-test was performed on the normalized ALFF maps with REST. The resultant statistical map was set at a combined threshold of *P*<0.005 and a minimum cluster size of 351 mm^3^ (13 voxels), which corresponded with the corrected threshold of *P*<0.05 determined by AlphaSim (http://afni.nih.gov/afni/docpdf/AlphaSim.pdf). For presentation purposes, the statistical maps were superimposed on the higher-resolution anatomical template available in REST and the MNI coordinates of peak *t*-value was transformed to Talarich coordinates with the REST Slice Viewer (a utility tool in REST) [32].

## Results

**Figure 1:**
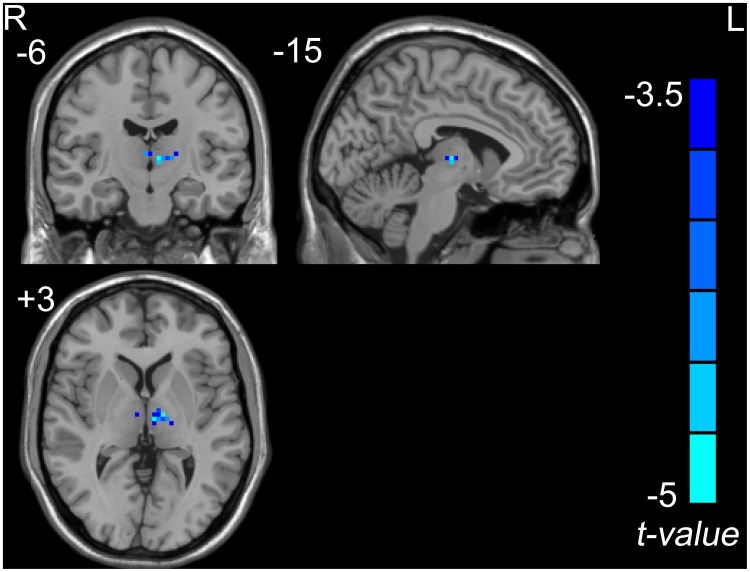
The decreased ALFF in the left thalamus in the simulated microgravity condition when combined with the normal condition (a combined threshold of *P*<0.005 and a minimum cluster size of 351 mm^3^ (13 voxels), which corresponded with the corrected threshold of *P*<0.05 determined by AlphaSim).

The ALFF results between the normal condition and a simulated microgravity condition are shown in Fig. 1 and Table 1. Compared to the normal condition, a decreased ALFF was observed in the left thalamus (including the medial dorsal nucleus and ventral lateral nucleus) in the simulated microgravity condition.

**Table 1:** The brain areas with significant ALFF differences between the simulated microgravity and normal conditions (a combined threshold of *P*<0.005 and a minimum cluster size of 351 mm^3^ (13 voxels), which corresponded with the corrected threshold of *P*<0.05 determined by AlphaSim).

Brain regions	Voxels size (mm^3^)	Talairach coordinates			Peak-t value
		X	Y	Z	
SMC < NC					
Left thalamus	540	−6	15	3	−6.0678
(including MD and VL)					

SMC: Simulated microgravity condition; NC: Normal condition; MD: Medial dorsal nucleus; VL: Ventral lateral nucleus.

## Discussion

Using EEG, a microgravity effect of an individual’s body fluid redistribution toward the head has been shown to have a strong influence on the SNA of the brain [14], [17]. Because of the low-resolution EEG limitations in space, we require the use of a resting-state fMRI to determine which brain areas reveal dysfunctions that may contribute to performance degradations. Previous studies have demonstrated that ALFF can reflect the extent of SNA; therefore, the altered ALFF with and without 72 h in a −6°HDT observed in our study is credible [20], [23], [24], [25], [26], [27], [31]. In our study, the decreased ALFF in the left thalamus (including the medial dorsal nucleus and ventral lateral nucleus) was observed with 72 h −6°HDT. This finding may reflect dysfunction in these areas in a simulated microgravity environment.

As a relay station for sensory information, the thalamus represents an irreplaceable role in cognitive processes. The thalamus is a sophisticated structure with several substructures that correspond to various cognitive functions, and the altered brain activities that occur in this area may lead to pervasive cognitive and behavioral impairments.

As an important part of thalamus, the medial dorsal nucleus has an osculating connection with the dorsal lateral prefrontal cortex, which is an important area for executive action and attention focus and is a supplementary motor area [33], [34], [35]. Hazlett et al. suggested that the medial dorsal nucleus integrates incoming sensory information with higher cortical regions that are involved in planning response strategies and play an important role in executive functions [36]. Similarly, Zoppelt et al. showed that executive dysfunction is associated with damage in the lateral section of the medial dorsal nucleus of the thalamus [37]. Altogether, we have reason to believe that the decreased ALFF observed in the medial dorsal nucleus of the thalamus in the present study may reflect an impairment of executive functions that is likely to contribute to human performance degradation in a microgravity environment.

The ventral lateral nucleus of the thalamus acts as a relay motor nucleus and is a nodal point in which the pathways from the cerebellum, vestibular nuclei, and main structures of the extra pyramidal system converge. Information is then further transmitted to the motor, premotor, and accessory motor regions of the brain cortex [38], [39], [40], [41]. In this sense, the ventral lateral nucleus of the thalamus potentially has an important role in motor control. A study on patients with unilateral asterixis showed that the impairment of the ventral lateral nucleus of the thalamus might interrupt the cerebellar input, thereby leading to motor dysfunction [42]. This finding confirms the above inferences from an alternate viewpoint. It is clear that the decreased ALFF observed in the ventral lateral nucleus of the thalamus in the present study may reveal a weakened function in this area that would affect the normal motor control in a negative way in a microgravity environment.

According to previous study [43], mental performance and brain activity of participants under microgravity may suffer from both direct effects of microgravity (e.g. shift of body fluid) on perceptual, cognitive, and psychomotor processes, and unspecific stress effects on these functions due to high workload sleep disturbances, or the general burden of adapting to the extreme living conditions in space. We mainly attribute the brain activity change in our study to direct effects of microgravity (e.g. shift of body fluid) while Schneider et al. mainly attribute the brain activity change in their study to mood change, the source of this difference is mainly due to the period of microgravity in our study is different with their study. In the study carried out by Schneider et al. [17], both period of stimulated weightlessness induced by parabolic flight and head down tilt bed rest are transitory (e.g. several seconds or several minutes) and environments changed frequently, which may induce acute stress to participants under such circumstances. As one of the major result caused by acute stress, mood change could induce hormone secretion increase which would in turn cause brain activity change. The brain activity change observed by Schneider et al. is most likely belonged to this case. In contrast, the period of microgravity in our study is longer, thus the effect caused by acute stress has largely reduced after 72 h HDT for the reason of adaption. Meanwhile, direct effects of microgravity (e.g. shift of body fluid) are still at work. With these reasons combined together, we have the reason to believe that the observed brain activity change in our study is mainly caused by direct effects of microgravity (e.g. shift of body fluid). Even so, factors such as fatigue and psychological conditions (e.g. inactivity induced mood change) really exist during HDT period according to previous researches. Kaname Hirayanagi et al. reported the prevalence of subjective fatigue in young healthy males during 14 days of −6° head-down bed rest [44], while Yuko Ishizaki et al. suggested depressive and neurotic levels were enhanced during bed rest period [45]. In this mean, head down tilt bed rest not only induce physiological change such as body fluid shift, but also result in subsequent psychological changes. The observed change of brain activity was not the result of body fluid shift alone, but the combined impact of physiological change and subsequent psychological changes.

The findings in the present study provide a new perspective to explain the occurrence of performance degradations in a microgravity environment. Based on the results of this study, to maintain an optimal performance in a microgravity environment, the use of dopaminergic agents to improve the local activities in the decreased areas may play a role. Although the results of this study are encouraging, limitations remain. Initially, because of technical limitations, the sampling rate (TR = 2 s) used in the data acquisition is low; we could not simultaneously record cardiac rate and respiratory rate [20]. As the BOLD signal is a complex function of changes in cerebral blood flow (CBF) and cerebral metabolic rate of oxygen consumption (CMRO_2_), the heart rate and respiratory rate are ought to been recorded as regressor to regress out the correlated artifacts. The lack of simultaneous physiological recording is indeed a flaw in the present study. Fortunately, there are some indirect evidences, to some extent, may embellish it. As we have known, previous studies of microgravity has demonstrated that CBF velocity increased initially in HDT and then deceased to baseline after 10 hour of HDT and gradually stabilized [46], [47]. That is, there ought to be no significant difference in CBF between the two conditions in the present study. What’s more, R.G. Wise et al. observed regional differences in the reactivity of the BOLD signal to CO_2_ in the grey matter. The BOLD signal in thalamus nearly has no change when concentration of CO_2_ varying [48]. The two evidences listed above may make the changed ALFF in thalamus observed in the present study seem to be a little more credible. But we should treat this flaw seriously and prevent the existence of the same flaw in the following studies. Secondly, the present study lacks a correlation analysis of cognitive test scores with our resting-state fMRI results. However, the cognitive functions corresponding to the thalamus have been examined multiple times in previous studies. The correlation analysis is, therefore, an unnecessary, repetitive work. In addition, participants’ activities were limited during HDT bed rest condition, it is hard to carry out motor tasks. Due to this limitation, we couldn’t provide data comparing brain function during motor or cognitive tasks between conditions. Even so, previous studies have propose that resting-state fMRI data can be used as an alternative to task fMRI data, which is of great importance when task fMRI data are not easily or accurately acquired [49]. In this mean, resting-state fMRI maybe is the appropriate method to detect the underlying neurophysiological mechanisms for cognitive impairments in weightlessness though this assertion is still need to be verified in the future. Third, the altered brain activities observed in the present study were obtained in a simulated microgravity environment. Indeed, there are differences between a simulated and real microgravity environment. Body fluid shift is just one of the multiple negative effects caused by microgravity, there are many other negative effects (such as radiation, altered circadian rhythm and fatigue et al.) that could not been stimulated by HDT. Fortunately, as a reliable stimulated model of microgravity, the body fluid shift effect caused by HDT could induce similar change in blood circulation as real microgravity, which may contribute to corresponding brain activity change at an overwhelming percent. Even so, we should remain cautious in extending the results to a real microgravity environment yet.

Taken together, this study’s observation of a decreased ALFF in the left thalamus (including the medial dorsal nucleus and ventral lateral nucleus) in a simulated microgravity condition is still an inspiring finding. Though there were several limitations required to be clarified in the future, it may be reasonable to speculate about the idea that a gravity change-induced redistribution of body fluid and subsequent psychological changes may disrupt the function of the left thalamus in the resting state, which may contribute to declined motor control abilities and executive functions in astronauts in a microgravity environment.
